# The Association between the School Environment and Adolescent Alcohol Drinking Behavior in Six Cities in China

**DOI:** 10.3390/nu15020368

**Published:** 2023-01-11

**Authors:** Ruixin Chi, Shijun Lu, Na Zhang, Man Zhang, Kaiwei Guo, Songming Du, Jing Guo, Guansheng Ma

**Affiliations:** 1 Department of Nutrition and Food Hygiene, School of Public Health, Peking University, 38 Xue Yuan Road, Haidian District, Beijing 100191, China; 2 Laboratory of Toxicological Research and Risk Assessment for Food Safety, Peking University, 38 Xue Yuan Road, Haidian District, Beijing 100191, China; 3 Institute of Food and Nutrition Development, Ministry of Agriculture and Rural Affairs, 12 Zhongguancun South Street, Haidian District, Beijing 100081, China; 4 Chinese Nutrition Society, 6 Guang An Men Nei Street, Xicheng District, Beijing 100053, China; 5 South Metropolitan Health Service, 14 Barry Marshall Parade, Murdoch, WA 6150, Australia

**Keywords:** food environment, alcohol drinking, school, adolescent, underage drinking

## Abstract

Drinking alcohol during adolescence has short-term and long-term effects on physical and mental health. At this stage, teenagers are greatly influenced by their schoolmates and friends. We conducted a multicenter cross-sectional study to investigate the association between school environment factors and adolescents’ drinking behavior in China. Using multistage stratified cluster sampling, the study investigated 27,762 middle school students from six cities in China. The logistic regression model was used to explore the association between the school environment and adolescent alcohol drinking behaviors after adjusting for confounders, including gender, age, city, location, and smoking status. Compared with students with none of their close friends drinking, students with more than half of their close friends drinking were more likely to drink in a year (OR = 20.148, 95% CI: 17.722–22.905, *p* < 0.001) and in a month (OR = 13.433, 95% CI: 11.779–15.319, *p* < 0.001). In addition, classmates’ drinking behaviors, friends’ persuasion, and attending parties were risk factors for adolescents’ drinking behavior, while the propaganda and regulations of banning drinking in school were protective factors. The school environment, especially friends drinking, is associated with students’ drinking behavior. It is necessary to mobilize the strength of schools and peers to strengthen the prevention and control of adolescent drinking.

## 1. Introduction

Alcohol consumption causes almost 3,000,000 deaths in the world per year. Drinking alcohol during adolescence has short-term and long-term effects on both physical and mental health [1]. Adolescence is a vital stage of growth and development. Healthy behaviors in this stage have an important impact on brain structure and function. It can cause a decline in cognitive and behavioral functions and academic performance and frequent injury events [2]. Moreover, alcohol consumption can affect physical and mental health throughout the lifetime [3,4]. A previous study showed that alcohol was one of the significant risk factors affecting people’s health, with ages ranging from 10 to 24 years old [5]. As a result, public health strategies should focus on how to limit adolescents’ alcohol consumption [6].

The World Health Organization reports that 43.0% of people over 15 years old are currently drinking alcohol, and 12.5% have previously consumed alcohol worldwide. A study showed that 51.1% of teenagers had consumed alcohol in China [7]. At present, the problem is becoming increasingly serious because the drinking rate is high among adolescents. The rate is increasing year by year [8]. Therefore, it is necessary to explore the risk factors related to drinking among adolescents. Recognition and control of these factors can help to regulate drinking behaviors [6,9].

Studies have shown that the food environment is associated with adolescents’ eating behaviors, e.g., calorie/fat intake [10,11,12,13,14]. Some studies have shown that family environmental factors have a certain impact on adolescent drinking behavior [15,16,17]. However, as adolescents age, peer influence increases, and family influence decreases [18]. In addition, drinking culture is prevalent in China, and some people believe that alcohol drinking is related to social skills, especially at parties [19]. Therefore, in China, socializing among friends has also become a reason for drinking [20,21,22]. Schools are essential places for teenagers to socialize. As a result, it is important to explore the association between school environment and adolescents’ drinking behaviors.

Some studies have also attempted to describe the school environment factors that influence adolescent drinking behavior, such as peers’ drinking behavior [23,24] and school education about the dangers of drinking [25]. However, the results were inconsistent across different studies. Moreover, variables related to the family environment are insufficient in studies. There is a lack of high-quality research results from China. As a result, the study hypothesized that school environmental factors were associated with adolescent drinking behavior in China. In addition, a multicity cross-sectional study was carried out to demonstrate the relationship between the school environment and adolescent drinking behavior in China.

## 2. Materials and Methods

### 2.1. Participants

We conducted a cross-sectional study in Beijing, Shanghai, and Guangzhou in 2013 and conducted the same investigation in Jinan, Chengdu, and Harbin in 2014. In select cities, we considered the willingness and difficulty of carrying out and organizing actual work and tried our best to choose cities with different representativeness of geographical regions and levels of economic development. These regions were the following: Northeast: Harbin; North: Beijing; East: Shanghai, Jinan; Southwest: Chengdu; South: Guangzhou. Among them, Beijing and Shanghai are municipalities directly under the central government. The other four cities (Harbin, Jinan, Chengdu, and Guangzhou) are provincial capitals. In this study, a multistage stratified cluster sampling method was adopted. According to geographical location, administrative division, and class size, 4–6 urban districts were randomly selected from the above six cities by random number table. Middle and high schools were selected randomly from the selected urban districts. All students in all three grades in each school were selected for a questionnaire survey. For the graduate grade (junior three, senior three) class, which could not be investigated in schools, an extra second grade (junior two or senior two) class was included for compensation. The Ethics Committee of the Chinese Center for Disease Control and Prevention approved the study, and informed consent was sought from students, parents, and schools prior to implementation. The ethical approval number of the study is 2013-019. Moreover, the researchers informed the subjects that they had the right to leave the study at any time. Under the guidance of the investigator, participants completed the questionnaires. After completing all questions and answers, they were compensated with a mechanical pencil.

### 2.2. Demographic Characteristics

Demographic characteristics were measured with a self-report questionnaire. Participants reported their gender, age, cities, and location via a general information questionnaire. Locations were divided into urban and suburban areas.

### 2.3. Assessment of Alcohol Drinking Behaviors

The Youth Risk Behavior Survey Questionnaire [26] and Global School-based Student Health Survey [27] are internationally accepted surveys of adolescent health behavior. Some items are related to alcohol consumption, for example, the number of drinking times in a certain period. According to these, we designed our questionnaires. We conducted a discussion with experts in related fields to create the final version of the questionnaire. In this study, the assessment of drinking behavior included having ever consumed alcohol, having consumed alcohol within a month, having consumed alcohol within a year and having ever been drunk. The results were collected as “yes” and “no”.

### 2.4. School Environment Related to Alcohol Drinking

School environment variables associated with alcohol consumption in this study included learning about the dangers of drinking alcohol in class, learning about the dangers of drinking on the school radio, board newspaper and propaganda board, whether there was a prohibition of alcohol in school, seeing their classmate drinking, having friends who persuade them to drink, the proportion of friends always drinking, and frequency of attending parties. The proportion of friends who were always drinking was divided into three categories: none, ≤50%, and >50%. The frequency of attending parties included four options: almost none, less than 1 time per week, and ≥1 times per week. Questionnaire of school environment related to alcohol drinking is shown in Appendix A.

### 2.5. Statistics

SAS 9.4 and SPSS 25.0 were used for data cleaning and analysis. We used SAS to clean the data. When there was a logical error in the data, it was set as a missing value. For example, if someone reported an age above 20, the record was set as a missing value. Because this situation was rare in middle-aged students in China, we could not validate this record. Meanwhile, if participants did not report their drinking behaviors, gender, age, smoking status or any variables above, they were excluded from the analysis. SPSS 25.0 was used to perform logistic regression analysis.

In this study, we set drinking behaviors as dependent variables (detailed information is in Section 2.3) and used all variables in 2.4 as independent variables in the analysis. The statistical approach was consistent with the methodology of our previous paper on family environment and student alcohol consumption [17]. However, in this study, we focused on school environment-related indicators that are particularly important to adolescents. The logistic regression model was used to analyze the relationship between the school environment and adolescent drinking behavior (Model 1). Confounders were determined by the literature and discussion with experts, including smoking status and demographic characteristics [28]. A model (Model 2) was analyzed after controlling for age, sex, age, city, location, and smoking. *p* < 0.05 was considered statistically significant.

### 2.6. Quality Control

All investigators received standardized workbooks and training. All the investigators had a medical education background and rich experience. At school, educators helped with the research. The questionnaires were collected using answer sheets and scanned by machines, and two researchers independently checked the data before forming the final database.

## 3. Results

### 3.1. Demographic Information

A total of 27,762 middle school students aged 12 to 20 were surveyed. The participants’ median age was 15.00, and the interquartile range was 3.00. In the study, the proportions of boys and girls were 48.3% and 51.7%, respectively, from urban and suburban areas. The basic demographic information of the research participants is shown in Table 1.

### 3.2. Alcohol Drinking Behavior

The survey results showed that 56.6% of the students surveyed had drunk alcohol, 44.8% had drunk alcohol in the past year, and 23.3% had drunk alcohol in the past month. A total of 19.3% of the students had never drunk alcohol. In another published study [17], we described drinking behaviors in detail among participants with different characteristics.

### 3.3. School Environment

The school environment factors related to drinking among the subjects are shown in Table 2, in which 46.5% of the subjects indicated that they had learned about the dangers of drinking in the course. Approximately 45.5% of the respondents said they had learned about the dangers of drinking through school broadcasts, bulletin boards, and billboards. A total of 76.5% of the students indicated that they would guide the school’s regulations on the prohibition of alcohol consumption. A total of 61.2% of students had seen their classmates drink. Those with no or less than half of their friends and those with more than half were 34.8%, 49.2%, and 16%, respectively. A total of 79.5% said they had been persuaded to drink by friends. A total of 42.9%, 51.3%, and 5.8% of the respondents rarely participated in queuing, less than once a month, 1–3 times a month, and more than once a week, respectively.

### 3.4. The Association between the School Environment and Adolescent Drinking Behavior

The school environment is closely related to adolescent drinking behavior. The logistic model shows the relationship between school environment variables and drinking behavior, as shown in Table 3. People who had friends who drank alcohol but less than 50% of the time were more likely to drink alcohol than ever have consumed alcohol in their lives (OR, odds ratio = 5.838, 95% CI, confidence interval: 5.478–6.223, *p* < 0.001; full adjustment: OR = 5.120, 95% CI: 4.785–5.479, *p* < 0.001), consumed alcohol within a year (OR = 6.571, 95% CI: 6.084–7.098, *p* < 0.001; full adjustments: OR = 5.734, 95% CI: 5.288–6.218, *p* < 0.001), and consumed alcohol within a month (OR = 5.490, 95% CI: 4.918–6.128, *p* < 0.001; full adjustment: OR = 4.988, 95% CI: 4.451–5.589, *p* < 0.001) than those whose friends did not drink alcohol. People with more than half of their friends who drank alcohol were 16.563 times more likely to have ever drank alcohol (OR = 22.762, 95% CI: 20.106–25.768, *p* < 0.001; full adjustment: OR = 16.563, 95% CI: 14.541–18.867, *p* < 0.001) and consumed alcohol within a year (OR = 27.747, 95% CI: 24.562–31.345, *p* < 0.001; full adjustment: OR = 20.148, 95% CI: 17.722–22.905) within a month (OR = 17.077, 95% CI: 15.077–19.342, *p* < 0.001; full adjustment: OR = 13.433, 95% CI: 11.779–15.319, *p* < 0.001) than those with none of their friends drinking. In addition, classmate drinking, friends urging drinking, and attending parties are risk factors for drinking behavior. To some extent, the propaganda and regulations of banning drinking in school are also protective factors for teenagers not to drink.

The school environment is also closely related to individual drunken behavior. The association logistic model shows the relationship between school environment variables and adolescent drinking behavior, as shown in Table 4. People whose friends drank were also more likely to get drunk than those whose friends did not. People with no more than half of their friends who drank were more likely to get drunk than those with none of their friends (OR = 5.520, 95% CI: 4.872–6.254, *p* < 0.001; full adjustment: OR = 4.301, 95% CI: 3.779–4.896, *p* < 0.001). People with more than half of their friends who drank were more likely to get drunk than those with none of their friends (OR = 16.614, 95% CI: 14.480–19.062, *p* < 0.001; full adjustment: OR = 10.176, 95% CI: 8.803–11.762, *p* < 0.001). In addition, drinking by classmates, persuading friends to drink, and participating in parties were all risk factors for getting drunk (*p* < 0.001). Publicity on campus about the dangers of drinking was a protective factor for drunkenness (*p* < 0.001).

## 4. Discussion

The study supports that school environment factors, including friends’ drinking behaviors and persuading, classmates’ drinking behaviors, and the frequency of attending parties, are related to adolescents’ drinking behaviors. This is consistent with other studies from other countries [23,24,29]. Currently, there are a lack of high-quality research results from China. The study provides evidence from China on this issue.

Several theories may explain the effect of the school environment on adolescent drinking behavior. Social learning theory holds that people adjust their behavior according to normative or typical behaviors [30]. For example, children are influenced by their peers and imitate their eating and exercise habits. Similarly, students may learn about the drinking behavior of their classmates or friends. Impression management theory argues that individuals change their behavior to maintain an image [31]. If drinking is prevalent among students, it is possible that some students will drink because they appear to fit in. Moreover, the social nature of drinking in China is related to food culture, so Chinese people should show respect and politeness when persuading people to drink. Tolerance of alcohol is an indication of courage to some extent [20,21,22].

The study also demonstrates that propaganda and regulations about the prohibition of drinking by schools are negatively correlated with adolescent drinking. Effective school propaganda on alcohol abstinence was a protective factor for adolescents in this study. However, there are currently inconsistencies in the influence of school environmental factors on drinking behaviors worldwide [25,29,32]. Some experiments showed that while campus propaganda could improve students’ knowledge about drinking, it did not affect students’ behavior. This study provides evidence from China on this issue. The difference between these results may be related to the propaganda’s intensity, effectiveness, and the characteristics of students.

Adolescent alcohol consumption has a significant impact on both physical and mental health at that time and in adulthood [3,4]. Therefore, it is vital to explore the influencing factors of adolescent drinking behavior. During adolescence, the influence of family gradually decreases and that of peers gradually increases [18]. Moreover, at this stage, teenagers have poor self-control and strong impulsivity [33]. Therefore, it is necessary to explore the school environmental factors of adolescent drinking in this study.

Most previous studies on the food environment have focused on the effects on calorie intake and vegetable and fruit consumption. In this area, there is a lack of research on drinking behavior [10]. Although some past studies have explored the relationship between the school environment and adolescent drinking behaviors, only a few factors have been identified. Worldwide, the relationship between the school food environment and adolescent drinking behavior is inconsistent. Not only in China but also worldwide, there is a high incidence at present [1,2]. Various school environmental factors are considered in this study at the same time. The friends’ drinking behaviors and persuading, classmates’ drinking behaviors, the frequency of attending parties, and the propaganda and regulations about the prohibition of drinking by schools are included in the model simultaneously, which is more detailed than other studies. Moreover, the adjusted model fully considered the influence of different cities, age, gender, urban area, and other factors. With a relatively large sample size, the results of this study represent the actual situation of a considerable number of Chinese teenagers, which is highly convincing. To the best of our knowledge, there is a lack of evidence from China about the drinking and school environment. This study suggests a relationship between the food environment and drinking behavior of Chinese students and their peers, which may play a specific reference role in future related intervention research and policymaking.

There are some limitations in this study. First, because of the limitation of data collection, some spatial school environmental factors and cause-and-effect relationships that exist between the existing situation, for example, stress from school, were not included [34]. Second, the survey was conducted through self-report, which was not accurate [35]. In addition, our research was conducted in six cities, representing Northeast, North, East, Southwest, and South China with better economic development, but some remote areas were not included. In that sense, it still cannot fully represent the whole situation in China. Moreover, in this study, we used drinking alcohol in the past month/year/ever (or not) and getting drunk as an assessment of alcohol drinking behaviors. Because of the study design, we did not collect quantitative alcohol consumption data. If there were quantitative indicators, the description of the results would be adequate and more detailed. Last but not least, as a cross-sectional survey conducted in 2013 and 2014, the study showed an association between school environmental factors and adolescents’ drinking behavior at that time, but it could not establish a causal relationship for temporality.

Schools are common places for adolescent health behavior intervention [36]. Studies have shown that the rate at which children start drinking alcohol rises mainly from the age of 10 [37]. School-based alcohol prevention programs are more effective starting in secondary school [38]. After understanding the school environment related to adolescent drinking, targeted supervision and control should be carried out to improve teenage health behavior at the whole school level and reduce the prevalence of drinking habits in school [39].

Further studies are also needed to verify the results and explore the causal relationship. In addition, it is important to explore suitable school environment assessment tools for adolescent drinking in China. Based on these works, more targeted interventions should be implemented. Appropriate interventions and policies should be designed and implemented according to health-related school environmental indicators to positively impact adolescent drinking behavior.

## 5. Conclusions

This study innovatively explored the relationship between the school food environment and adolescent drinking behavior in China and identified factors closely related to drinking behavior. Moreover, it is significant for the targeted prevention and control of adolescent drinking in China.

## Figures and Tables

**Table 1:** The demographic information of participants.

		N	%
Sex			
	Men	13,417	48.3
	Women	14,345	51.7
Age			
	18~20	3012	10.8
	15~17	14,516	52.3
	12~14	10,234	36.9
City			
	Beijing	3782	13.6
	Shanghai	3142	11.3
	Guangzhou	5241	18.9
	Jinan	4889	17.6
	Chengdu	5363	19.3
	Harbin	5345	19.3
Location			
	Suburb	13,387	48.2
	Urban	14,375	51.8
Total		27,762	100.0

**Table 2:** School environment regarding alcohol drinking.

		N	%
Learning about the dangers of drinking alcohol in class			
	Yes	12,794	46.5
	No	14,695	53.5
Learning about the dangers of drinking on school radio, board newspaper and propaganda board			
	Yes	12,415	45.5
	No	14,877	54.5
Prohibition of alcohol in school			
	Yes	20,864	76.5
	Do not know	6402	23.5
Seeing my classmate drinking			
	Yes	16,574	61.2
	No	10,511	38.8
Some of my best friends drinking			
	None	9583	34.8
	≤50%	13,560	49.2
	>50%	4397	16
My friends persuading me to drink			
	Yes	21,597	79.5
	No	5582	20.5
Frequency of attending parties			
	Almost no	11,770	42.9
	<1 time per week	14,068	51.3
	≥1times per week	1594	5.8

**Table 3:** Unadjusted and adjusted logistic regression analyses of school environment factors and alcohol drinking.

	Model 1						Model 2					
	B	Wald	*p*	OR	95% CI		B	Wald	*p*	OR	95% CI	
					Lower	Upper					Lower	Upper
Ever consumed alcohol												
Learning in class	0.090	6.924	0.009 *	1.094	1.023	1.169	0.088	6.040	0.014 *	1.092	1.018	1.172
Learning in school (without class)	0.305	79.719	0.000 *	1.356	1.268	1.450	0.232	42.187	0.000 *	1.261	1.176	1.353
Prohibition of alcohol in school	0.032	0.711	0.399	1.032	0.959	1.111	0.073	3.379	0.066	1.076	0.995	1.163
Seeing my classmates drinking	0.246	61.360	0.000 *	1.279	1.203	1.361	0.132	15.893	0.000*	1.142	1.070	1.218
Some of my friends drinking (0 = ref.)												
≤50%	1.764	2941.076	0.000 *	5.838	5.478	6.223	1.633	2236.366	0.000 *	5.120	4.785	5.479
>50%	3.125	2437.566	0.000 *	22.762	20.106	25.768	2.807	1785.153	0.000 *	16.563	14.541	18.867
My friends persuade myself drinking	−0.298	56.594	0.000 *	0.742	0.686	0.802	−0.195	21.284	0.000 *	0.823	0.758	0.894
The frequency of attending parties (Almost no = ref.)												
<1 time per week	0.671	473.149	0.000 *	1.957	1.842	2.079	0.581	315.469	0.000 *	1.787	1.676	1.905
≥1times per week	0.331	24.852	0.000 *	1.392	1.222	1.585	0.254	13.233	0.000 *	1.289	1.124	1.477
Consumed alcohol within a year												
Learning about the dangers in class	0.016	0.193	0.661	1.017	0.945	1.094	0.012	0.100	0.752	1.012	0.938	1.093
Learning about the dangers in school (Without class)	0.254	45.958	0.000 *	1.290	1.198	1.388	0.196	25.213	0.000 *	1.217	1.127	1.314
Prohibition of alcohol in school	0.145	13.037	0.000 *	1.156	1.069	1.251	0.160	14.192	0.000 *	1.173	1.080	1.274
Seeing my classmates drinking	0.256	57.768	0.000 *	1.292	1.209	1.380	0.163	21.254	0.000 *	1.178	1.099	1.262
Some of my friends drinking (0 = ref.)												
≤50%	1.883	2290.314	0.000 *	6.571	6.084	7.098	1.746	1788.211	0.000 *	5.734	5.288	6.218
>50%	3.323	2853.278	0.000 *	27.747	24.562	31.345	3.003	2105.252	0.000 *	20.148	17.722	22.905
My friends persuade myself drinking	−0.308	54.780	0.000 *	0.735	0.677	0.797	−0.195	19.488	0.000 *	0.823	0.754	0.897
The frequency of attending parties (Almost no = ref.)												
<1 time per week	0.982	832.408	0.000 *	2.669	2.497	2.853	0.903	635.445	0.000 *	2.468	2.300	2.647
≥1times per week	0.583	63.498	0.000 *	1.791	1.552	2.067	0.501	42.965	0.000 *	1.651	1.421	1.917
Consumed alcohol within a month												
Learning about the dangers in class	−0.095	5.775	0.016 *	0.909	0.841	0.983	−0.064	2.397	0.122	0.938	0.865	1.017
Learning about the dangers in school (Without class)	0.115	8.227	0.004 *	1.122	1.037	1.213	0.056	1.833	0.176	1.058	0.975	1.147
Prohibition of alcohol in school	0.235	33.153	0.000 *	1.265	1.168	1.370	0.240	31.662	0.000 *	1.271	1.169	1.382
Seeing my classmates drinking	0.289	69.329	0.000 *	1.334	1.247	1.428	0.242	44.836	0.000 *	1.274	1.187	1.367
Some of my friends drinking (0 = ref.)												
≤50%	1.703	920.518	0.000 *	5.490	4.918	6.128	1.607	765.711	0.000 *	4.988	4.451	5.589
>50%	2.838	1994.500	0.000 *	17.077	15.077	19.342	2.598	1501.712	0.000 *	13.433	11.779	15.319
My friends persuade myself drinking	−0.272	46.826	0.000 *	0.762	0.705	0.823	−0.181	18.680	0.000 *	0.834	0.768	0.906
The frequency of attending parties (Almost no = ref.)												
<1 time per week	0.922	549.655	0.000*	2.514	2.328	2.716	0.846	430.608	0.000 *	2.331	2.152	2.524
≥1times per week	0.891	142.377	0.000*	2.438	2.106	2.822	0.813	111.005	0.000 *	2.254	1.938	2.622

* *p* < 0.05. OR: odds ratio. CI: confidence interval. Model 1: nonadjusted model. Model 2: adjusted for age, gender, age, city, and location.

**Table 4:** Unadjusted and adjusted logistic regression analyses of school environment factors and getting drunk.

	Model 1						Model 2					
	B	Wald	*p*	OR	95% CI		B	Wald	*p*	OR	95% CI	
				Lower	Upper					Lower	Upper	
Learning about the dangers in class	−0.050	1.387	0.239	0.951	0.876	1.034	−0.067	2.238	0.135	0.935	0.857	1.021
Learning about the dangers in school (Without class)	0.226	27.874	0.000 *	1.253	1.152	1.362	0.146	10.577	0.001 *	1.157	1.060	1.263
Prohibition of alcohol in school	−0.128	8.161	0.004 *	0.880	0.806	0.961	−0.069	2.111	0.146	0.933	0.850	1.024
Seeing my classmates drinking	0.309	70.202	0.000 *	1.362	1.267	1.464	0.190	23.509	0.000 *	1.209	1.120	1.306
Some of my friends drinking (0 = ref.)												
≤50%	1.708	718.620	0.000 *	5.520	4.872	6.254	1.459	488.295	0.000 *	4.301	3.779	4.896
>50%	2.810	1605.668	0.000 *	16.614	14.480	19.062	2.320	984.952	0.000 *	10.176	8.803	11.762
My friends persuade myself drinking	−0.581	205.516	0.000 *	0.559	0.516	0.605	−0.457	110.943	0.000 *	0.633	0.581	0.689
The frequency of attending parties (Almost no = ref.)												
<1 time per week	0.611	213.265	0.000 *	1.842	1.697	1.999	0.489	123.203	0.000 *	1.631	1.496	1.778
≥1times per week	0.609	59.058	0.000 *	1.839	1.575	2.149	0.567	46.146	0.000 *	1.763	1.497	2.076

Notes: * *p* < 0.05. OR: odds ratio. CI: confidence interval. Model 1: nonadjusted model. Model 2: adjusted for age, gender, age, city, and location.

## Data Availability

Not applicable.

## Appendix A

**Table A1:** Questionnaire of school environment related to alcohol drinking.

Item	Option
Learning about the dangers of drinking alcohol in class	
	Yes
	No
Learning about the dangers of drinking on school radio, board newspaper and propaganda board	
	Yes
	No
Prohibition of alcohol in school	
	Yes
	Do not know
Seeing my classmate drinking	
	Yes
	No
Some of my best friends drinking	
	None
	≤50%
	>50%
My friends persuading me to drink	
	Yes
	No
Frequency of attending parties	
	Almost no
	<1 time per week
	≥1 times per week
